# NET1 promotes HCC growth and metastasis *in vitro* and *in vivo* via activating the Akt signaling pathway

**DOI:** 10.18632/aging.202845

**Published:** 2021-04-11

**Authors:** Zhen-Hua Chen, Qian-Zhi Ni, Xiu-Ping Zhang, Ning Ma, Jing-Kai Feng, Kang Wang, Jing-Jing Li, Dong Xie, Xing-Yuan Ma, Shu-Qun Cheng

**Affiliations:** 1 Department of Hepatic Surgery VI, Eastern Hepatobiliary Surgery Hospital, Second Military Medical University, Shanghai 200433, China; 2 Department of General Surgery, Zhejiang Provincial Armed Police Corps Hospital, Hangzhou 310051, Zhejiang Province, China; 3 State Key Laboratory of Bioreactor Engineering, East China University of Science and Technology, Shanghai 200237, China; 4 CAS Key Laboratory of Nutrition, Metabolism and Food Safety, Shanghai Institute of Nutrition and Health, Shanghai Institutes for Biological Sciences, University of Chinese Academy of Sciences, Chinese Academy of Sciences, Shanghai 200031, China

**Keywords:** NET1, growth, metastasis, hepatocellular carcinoma, Akt

## Abstract

Neuroepithelial cell transforming gene 1 (NET1), a member of the guanine nucleotide exchange factor family, is involved in various cancers, including gastric cancer, breast cancer and glioma. However, the role of NET1 in hepatocellular carcinoma (HCC) remains largely uncovered. In this study, we found that NET1 expression was upregulated in HCC, and that upregulated NET1 expression was closely associated with poor prognosis and some clinical characteristics in HCC patients. Whilst forced expression of NET1 in HCC cells was observed to significantly promote cell growth and metastasis *in vitro* and *in vivo*; downregulation of NET1 was shown to exhibit an opposite inhibitory effect. RNA-seq analysis and gene set enrichment analysis demonstrated that knockdown of NET1 significantly suppressed the level of Akt phosphorylation level and the expression of Akt downstream genes in HCC cells. Moreover, MK2206, a potent Akt inhibitor was shown to block the NET1-induced effects in HCC. Taken together, this study demonstrated that, through the Akt signaling pathway, NET1 plays an oncogenic role in HCC progression and metastasis. Hence, NET1 may potentially be used as a potential therapeutic target and prognostic marker of HCC.

## INTRODUCTION

Hepatocellular carcinoma (HCC) is the fifth most frequent tumor and the second leading cause of cancer-related mortality [1, 2]. Despite recent advancements of multiple therapy approaches, such as surgery, transarterial chemoembolization and radiotherapy, the prognosis of HCC remains relatively poor due to the higher tendency of recurrence (5-year recurrence rate >70%) [3, 4]. To provide some new insights into the treatment of HCC, it is of utmost urgency to unravel the molecular mechanism of HCC progression and metastasis.

Neuroepithelial cell transforming gene 1 (NET1) is a guanine-nucleotide exchange factor (GEF) family member, and is able to activate and regulate members of the Rho family [5, 6]. Some GEFs, such as ASEF, Bcr and GEFH1 have been shown to be highly responsible for the development of multiple cancers [7–9]. Meanwhile, previous studies have shown that NET1 is required for cell motility and extracellular matrix (ECM) invasion [10, 11]. NET1 has been found to be induced by lysophosphatidic acid (an activator of RhoA) in order to promote cell invasion, cell migration, and cytoskeletal actin organization in gastric cancer [12]. NET1 has been discovered as a significant indicator of poor clinical prognosis of several cancers, including breast cancer, glioma and adenocarcinoma of the oesophagogastric junction (AOG) [13–15]. Moreover, NET1 has been demonstrated to significantly enhance B-cell acute lymphoblastic leukemia cell proliferation and resistance to doxorubicin [16]. Although the clinicopathological roles of NET1 in HCC have been explored in a preliminary study [17], the detailed biological functions and molecular mechanisms of NET1 in the regulation of HCC proliferation and metastasis have not been fully elucidated using *in vitro* and *in vivo* approaches.

In this work, we observed that NET1 expression was overexpressed in human HCC tissue and was markedly associated with prognostic significance and clinicopathological parameters. We further demonstrated that NET1 promoted the proliferation and metastasis of HCC tumor *in vitro* and *in vivo*. Our RNA-seq analysis indicated that NET1 significantly promotes the Akt pathway and the metabolism of nitrogen, glycine, serine and threonine.

Taken together, our findings reveal the functions and mechanisms of NET1 in HCC growth and metastasis, indicating that NET1 can be a promising treatment target for HCC.

## MATERIALS AND METHODS

### Reagents

Mouse monoclonal antibody against NET1 (cat. no. sc-271941) was purchased from SantaCruz. Anti-Akt (cat. no. 4685) and phospho-(p) Akt (Ser473; cat. no. 4060) antibodies were supplied by Cell Signaling Technology. Horseradish peroxidase-conjugated secondary antibody and anti-GAPDH (cat. no.60004-1-Ig) antibody were supplied by Proteintech. MK2206 (cat. no. HY-10358), a potent Akt inhibitor, was purchased from MCE.

### HCC specimens and tissue microarray analysis

RNA samples were extracted from 45 pairs of primary HCC and adjacent normal tissues, followed by real-time PCR analysis; while protein samples were isolated from 12 pairs of tissue samples, followed by Western blotting. All tissue samples were retrieved from the Eastern Hepatobiliary Surgery Hospital (EHBH, Shanghai, China), and were were snap-frozen immediately after surgical procedure. Tissue microarray (TMA) was conducted on 210 paraffin-embedded primary HCC and paired normal tissues at EHBH. This research was approved by the Ethics Review Committee of EHBH, and was carried out in compliance with the Declaration of Helsinki. All participants signed an informed consent document before enrolment.

### Immunohistochemistry (IHC)

Paraffin-embedded tissue sections were deparaffinized and rehydrated before being subjected to endogenous peroxidase inhibition. The sections were rinsed thrice in PBS (0.01 mol/l), and blocked with PBS (0.01 mol/l) containing 5% BSA and 0.3% Triton X-100 for 1 hour. After incubation with anti-NET1 at 4°C overnight, the samples were incubated again with the secondary antibody at room temperature for 2 hours. After being developed with 3,3’-diaminobenzidine (0.03%) and H_2_O_2_ (0.003%) in Tris-HCl (0.05 mol/l, pH 7.6), the extent and staining intensity examined automatically by Vectra 2 system (PerkinElmer, USA) were calculated using H-score as previously described [18]. According to the median score of NET1, tissue samples with final H-scores of <81 and ≥81 were categorized as NET1 “low” and “high” expression, respectively.

### RNA isolation and real-time PCR

Total RNA was isolated from HCC cells and tissue specimens using TRIzol reagent (Invitrogen). Two micrograms of RNA extracts were reverse-transcribed into cDNA using the PrimeScript RT reagent Kit (Takara) as previously described [19]. SYBR Premix Taq (Yeasen Biotech) was used for real-time PCR analysis. The primers used are presented in Supplementary Table 1.

### RNA sequencing

RNA samples from 5 duplicates of NET1-depleted YY-8103 and scrambled cells were subjected to RNA sequencing. The limma package (Version 3.34.7, https://bioconductor.org/packages/release/bioc/html/limma.html) coupled with R software (http://www.R-project.org) for selected for data analysis.

### Cell culture

Human HCC cell lines (LM3, Huh7, Hep3B and YY-8103) were supplied by the Chinese Academy of Sciences Collection Committee cell bank (Shanghai, China). CSQT-2 cell line was set up in our laboratory. All cells were grown in DMEM medium (Gibco) containing 1% penicillin/streptomycin (Sangong Biotech) and 10% FBS (Anlite), and maintained at 37°C in a humidified incubator with 5% CO_2_.

### Plasmids and stable cell lines

Full-length cDNA encoding human NET1 was amplified by RT-PCR using human whole blood samples. NET1 expression vector construct was generated by cloning the amplified NET1 cDNA into p23-3×flag-GFP plasmid. Lentiviral short hairpin RNA (shRNA) plasmids for NET1 were designed based on the Qiagen’s software (Valencia, CA, USA). Next, HCC cell lines were infected with packaged lentivirus, and then subjected to GFP sorting or puromycin (4 μg/mL) treatment a day later. NET1 shRNA sequence pairs are listed in Supplementary Table 1.

### Western blot

The tissue samples were lysed in RIPA Lysis Buffer and PMSF (Thermo Scientific, USA) by following the manufacturer's instructions. After centrifugation (10,000 g, 15 minutes, 4°C), the concentrations of protein samples were assessed using the Bradford reagent (Sigma). Then, 20 μg of protein were separated on 10% SDS-PAGE, and transferred onto PVDF membranes. After blocking with 5 % skim milk for 1 hour at room temperature, the membranes were immunoblotted with the indicated primary antibodies at 4°C for 24 hours, and immunoblotted again with the corresponding secondary antibody solution at room temperature for 2 hours. Lastly, the immunoblots were detected with enhanced chemiluminescent reagent (Pierce).

### MTT assays

*In vitro* cell growth was evaluated by MTT assay. The cells (1 × 10^3^ cells/well) were grown on a 96-well plate, and their viability was detected by adding 20 μl of MTT (5 mg/ml) after 1–7 days of incubation. After a further incubation step at 37°C for 4 hours, the cell medium was aspirated, and the cells were rinsed in PBS and added with 200 μl DMSO under gentle agitation. Optical density (OD) values were recorded at 490nm using a microplate reader.

### Crystal violet assays

The cells (1,000 cells/well) were grown on 6-well dishes containing culture medium and 10% FBS. The culture medium was changed every 3 days. Following 14 days of incubation, the cells were subjected to crystal violet staining. Lastly, OD values were assessed at 570 nm using the microplate reader.

### Transwell assays

HCC cell invasion and migration analysis was carried out using a 24-well Transwell chamber (8-μm pore size; Corning, NY, USA). Briefly, cell suspension (150 μL, 1 × 10^5^ cells) was positioned into the top chamber with FBS-free medium, whereas 500 μL medium with 10% FBS was positioned into the bottom chamber. For the invasion analysis, Matrigel (50 μl; BD Biosciences) was used to precoat the membrane surface. After incubation at 37°C for 24 hours, the invading cells were fixed with methanol and then stained with crystal violet (0.1%) for 10 minutes at room temperature. The cells in 5 randomly chosen fields were counted under an optical microscope (Olympus Corp.) at ×200 magnification.

### Animal studies

The animal experimentation was approved by the Animal Ethical and Welfare Committee of the Shanghai Institutes for Nutritional Sciences, Chinese Academy of Sciences. For orthotopic xenograft experiments, 100 μL of HCC cell suspension (1 × 10^6^ cells/mouse) was infused into the right flanks of nude mice, followed by close monitoring. Tumor volume was measured each week based on the following equation: (length × width^2^) × 0.5. Five weeks later, tumor tissues were excised from the sacrificed mice, weighed and photographed.

For intrahepatic metastasis assays, the cells labeled with luciferase (5 × 10^5^ cells/mouse) were infused into the left liver of nude mice. For lung metastasis assays, cells (1 × 10^6^ cells/mouse) were infused into the tail veins of nude mice. Metastatic lesions were monitored weekly based on luciferase expression levels. Mice were intraperitoneally infused with aqueous solutions of D-luciferin before being anaesthetized by isoflurane and imaged with an IVIS imaging system (Xenogen). Six or eight weeks later, mice were euthanized, and their livers were photographed and the foci numbers were counted. For survival assays, cells (5 × 10^5^ cells/mouse) were infused into the left liver of nude mice, which were then raised under standard conditions until death. The time to death in mice was recorded within 60 days.

### Statistical analyses

All statistical tests were conducted using SPSS version 20.0 software (Chicago, IL, USA) and GraphPad Prism version 5.0 (CA, USA). The comparisons among groups were performed with Student’s *t*-test or ANOVA and Tukey post hoc test. Association between clinicopathological characteristics and NET1 expression level was evaluated with Chi-squared test. Disease-free survival (DFS) and overall survival (OS) were evaluated based on Kaplan-Meier curves, and the results were matched with those generated by log-rank tests. Independent predictors of OS and DFS were detected using univariate and multivariate analyses with step-wise selection. Cox proportional hazards model was employed to determine the significant prognostic factors in univariate analysis. The level of statistical significance was set at 0.05.

### Data availability information

All data generated or analyzed in this study are included in this published article.

## RESULTS

### Association between NET1 expression and HCC

**Figure 1:**
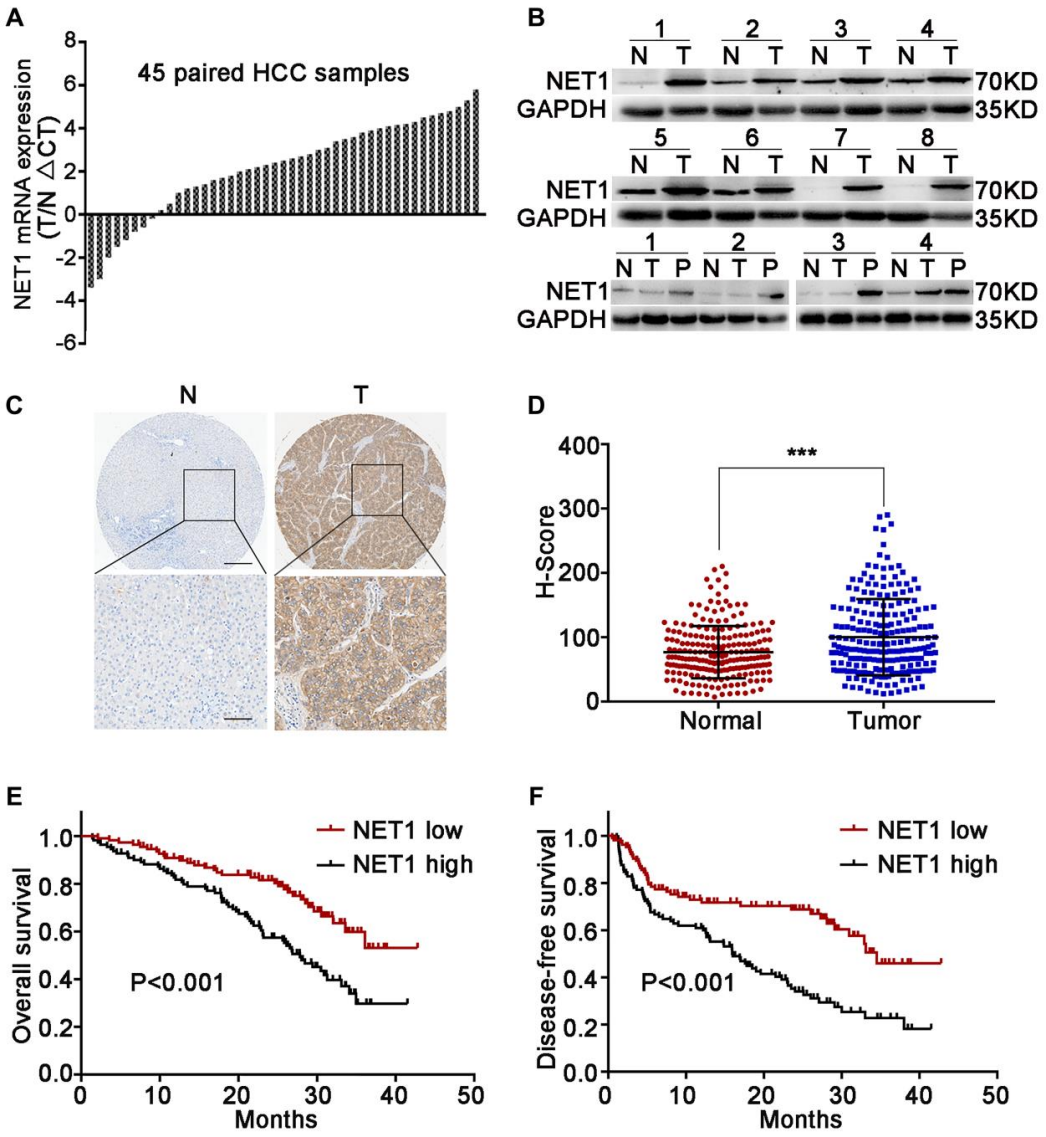
**Upregulation of NET1 in HCC tissues is associated with clinical prognosis.** (**A**) NET1 mRNA levels in 45 pairs of N and T tissues were determined by real-time PCR. Expression of NET1 was normalized to that of GAPDH. (**B**) NET1 protein levels in 8 pairs of N and T tissues, and in 4 pairs of N, T and P tissues were determined by Western blot; GAPDH was used as a loading control. (**C**) Immunohistochemistry staining of NET1 in paired N and T tissues from two patients. Scale bars, 250 μm; 100 μm. (**D**) H-scores of NET1 staining intensity in N (*n* = 210) and T (*n* = 210) tissues. (**E** and **F**) Kaplan–Meier analysis of overall survival (**E**) and disease-free survival (**F**) using tissue microarray (TMA) data of 210 patients. N, non-carcinoma normal tissues; T, tumor tissues; P, portal vein tumor thrombus tissues. ^***^P < 0.001.

Firstly, the mRNA levels of NET1 in 45 pairs of clinical HCC and paired normal tissues were evaluated. NET1 was upregulated in 37 pairs of tissues, which accounted for 82% of total samples (Figure 1A). Next, the protein levels of NET1 were detected in 12 pairs of randomly selected HCC tissues and the corresponding matched normal tissues. Elevated protein expression of NET1 was noted in all tested paired tissues (Figure 1B). The relationship between NET1 expression and some clinical features was examined based on a TMA constructed from 210 HCC specimens by IHC staining. Immunohistochemistry analysis revealed that the H-score of NET1 staining in tumor tissue was remarkably higher that of the adjacent normal tissue (*P* < 0.001; Figure 1C, 1D). In addition, HCC patients with high NET1 expression (with H-score ≥ 81) were found to exhibit poor DFS and OS (both *P* < 0.001) compared to those with low NET1 expression (H-score <81; Figure 1E, 1F). Collectively, these results revealed that NET1 is overexpressed in HCC tissue, and that increased NET1 expression may confer poor clinical prognosis in HCC patients.

Next, the association between NET1 expression and clinicopathologic characteristics in 210 HCC patients was explored. As summarized in Supplementary Table 2, high NET1 expression was found to be associated with large tumor size (*P* = 0.011), high alpha-fetoprotein (AFP) level (*P* = 0.015), micro metastasis (*P* = 0.004), portal vein tumor thrombus (PVTT, *P* = 0.025) and histologic evidence of microvascular invasion (*P* = 0.025). Cox multivariate analysis revealed several risk factors for OS (Supplementary Table 3) as follows: multiple tumors (*P* < 0.001), AFP ≥400 ng/ml (*P* = 0.001), tumor diameter ≥5 cm (*P* = 0.022), microvascular invasion (*P* = 0.003) and high NET1 expression (*P* = 0.007). The risk factors for DFS are shown as follows (Supplementary Table 4): presence of micro metastasis (*P* = 0.011) and high NET1 expression (*P* = 0.003).

### NET1 overexpression induces the growth, migration, and invasion and of HCC cells

**Figure 2:**
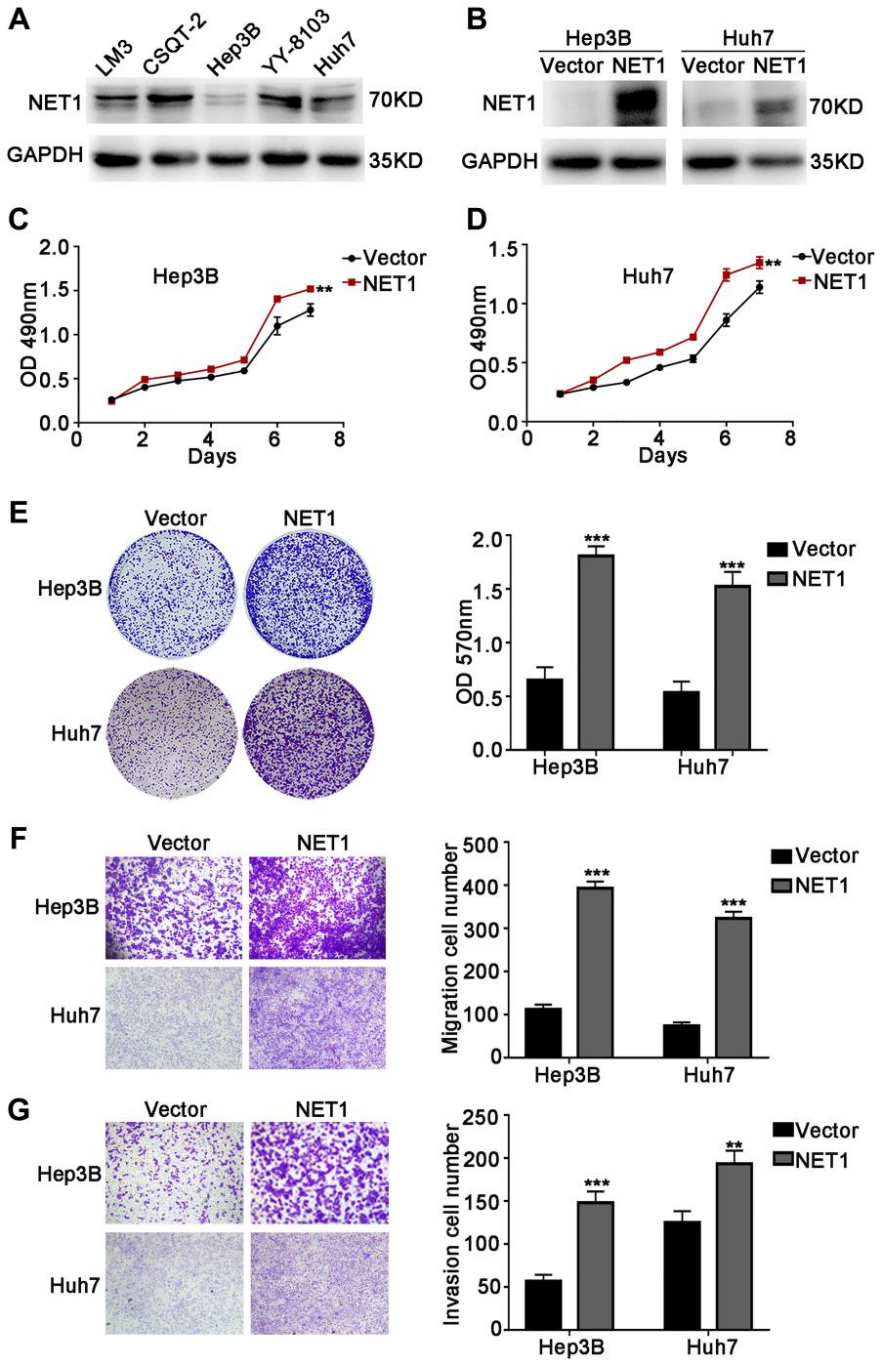
**NET1 overexpression promotes HCC cell proliferation, migration and invasion *in vitro***. (**A**) Western blot analysis of NET1 expression in 5 HCC cell lines. (**B**) Western blot analysis of NET1 overexpression efficiency in HCC cell lines (Hep3B and Huh7). (**C** and **D**) MTT assays showing the effects of NET1 overexpression on the proliferation of Hep3B (**C**) and Huh7 (**D**) cells. (**E**) Crystal violet assays showing the effects of NET1 overexpression on the proliferation of Hep3B and Huh7 cells. Left panel: crystal violet assay. Right panel: OD values of crystal violet assays. (**F** and **G**) Transwell assays showing the effects of NET1 overexpression on the migration (**F**) and invasion (**G**) of Hep3B and Huh7 cells. Left panel: transwell assays. Right panel: calculation of cells that have migrated (**F**) or invaded (**G**) through the filter following eosin staining. All data are presented as mean ± SE. ^**^P < 0.01, ^***^P < 0.001 vs vector cells.

The above research findings encourage us to test the biological roles of NET1 in HCC growth and metastasis *in vitro*. Firstly, NET1 expression levels were evaluated in several HCC cell lines (Figure 2A). NET1 was observed to be stably overexpressed in Hep3B and Huh7 cells, which had relatively low expression of NET1. The overexpression of NET1 protein in the two cell lines was also verified by Western blotting (Figure 2B). Then, the impacts of NET1 on HCC cell growth were analyzed by MTT (Figure 2C, 2D) and crystal violet (Figure 2E) assays. Our results showed that high NET1 expression markedly induced the proliferation and colony formation of Hep3B and Huh7 cells. Furthermore, transwell assays demonstrated that high NET1 expression remarkably increased the migration and invasion capabilities of Hep3B and Huh7 cells (Figure 2F, 2G). Taken together, the results above indicated that NET1 overexpression induced the proliferation, migration and invasion of HCC cell lines.

### NET1 knockdown inhibits the proliferation, migration, and invasion of HCC cells

**Figure 3:**
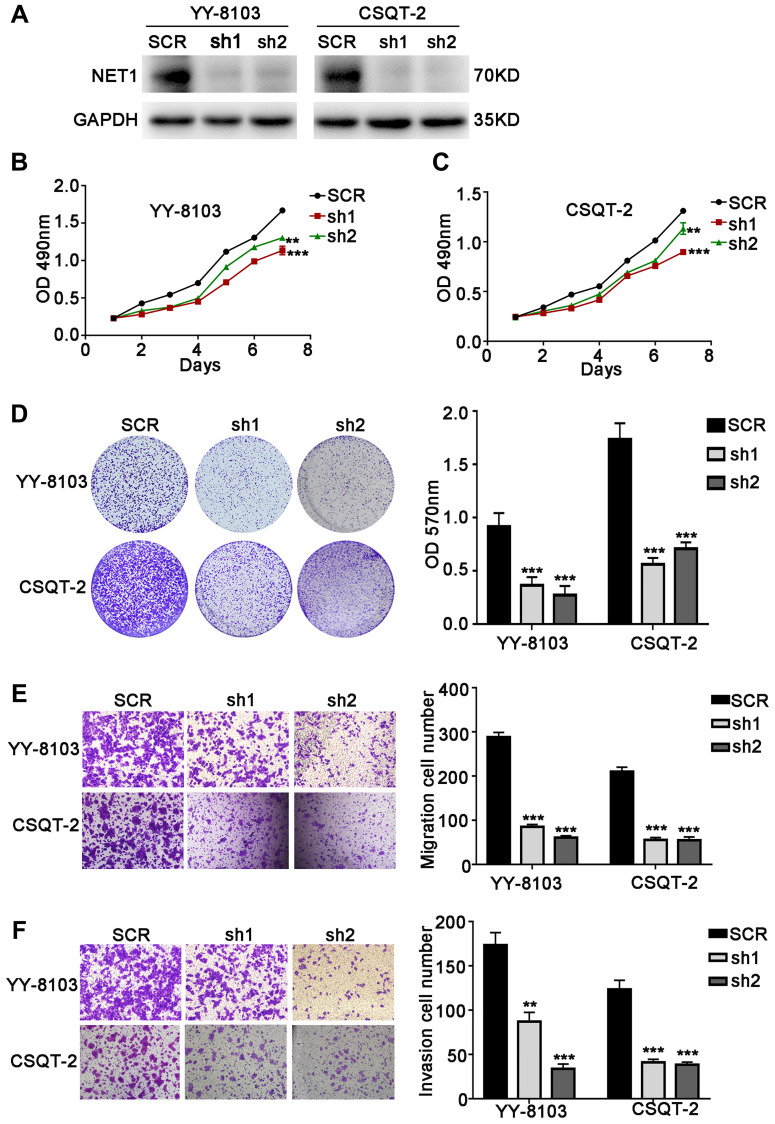
**NET1 knockdown inhibits HCC cell proliferation, migration and invasion *in vitro*.** (**A**) Western blot analysis showing the knockdown efficiency of NET1 in HCC cell lines (YY-8103 and CSQT-2). (**B** and **C**) MTT assays showing the effects of NET1 knockdown on the proliferation of YY-8103 (**B**) and CSQT-2 (**C**) cells. (**D**) Crystal violet assays showing the effects of NET1 knockdown on the proliferation of YY-8103 and CSQT-2 cells. Left panel: crystal violet assays. Right panel: OD values of crystal violet assays. (**E** and **F**) Transwell assays showing the effects of NET1 knockdown on the migration (**E**) and invasion (**F**) of YY-8103 and CSQT-2 cells. Left panel: transwell assays. Right panel: calculation of cells that have migrated (**E**) or invaded (**F**) through the filter following eosin staining. All data are presented as mean ± SE. ^**^P < 0.01, ^***^P < 0.001 vs SCR cells.

To further confirm the functions of NET1, its expression levels were knocked down by 2 independent shRNAs in YY-8103 and CSQT-2 cells with relatively high NET1 expression (Figure 3A). Based on MTT (Figure 3B, 3C) and crystal violet assays (Figure 3D), we observed that NET1 knockdown inhibited the proliferation and colony formation of YY-8103 and CSQT-2 cells. Moreover, transwell assay demonstrated that NET1 downregulation markedly suppressed the invasion and migration capabilities of the two cells (Figure 3E, 3F). Altogether, these findings indicated that downregulation of NET1 expression may exert a protective effect by suppressing the proliferation, migration and invasion of HCC cell lines.

### NET1 induces HCC oncogenesis and metastasis

**Figure 4:**
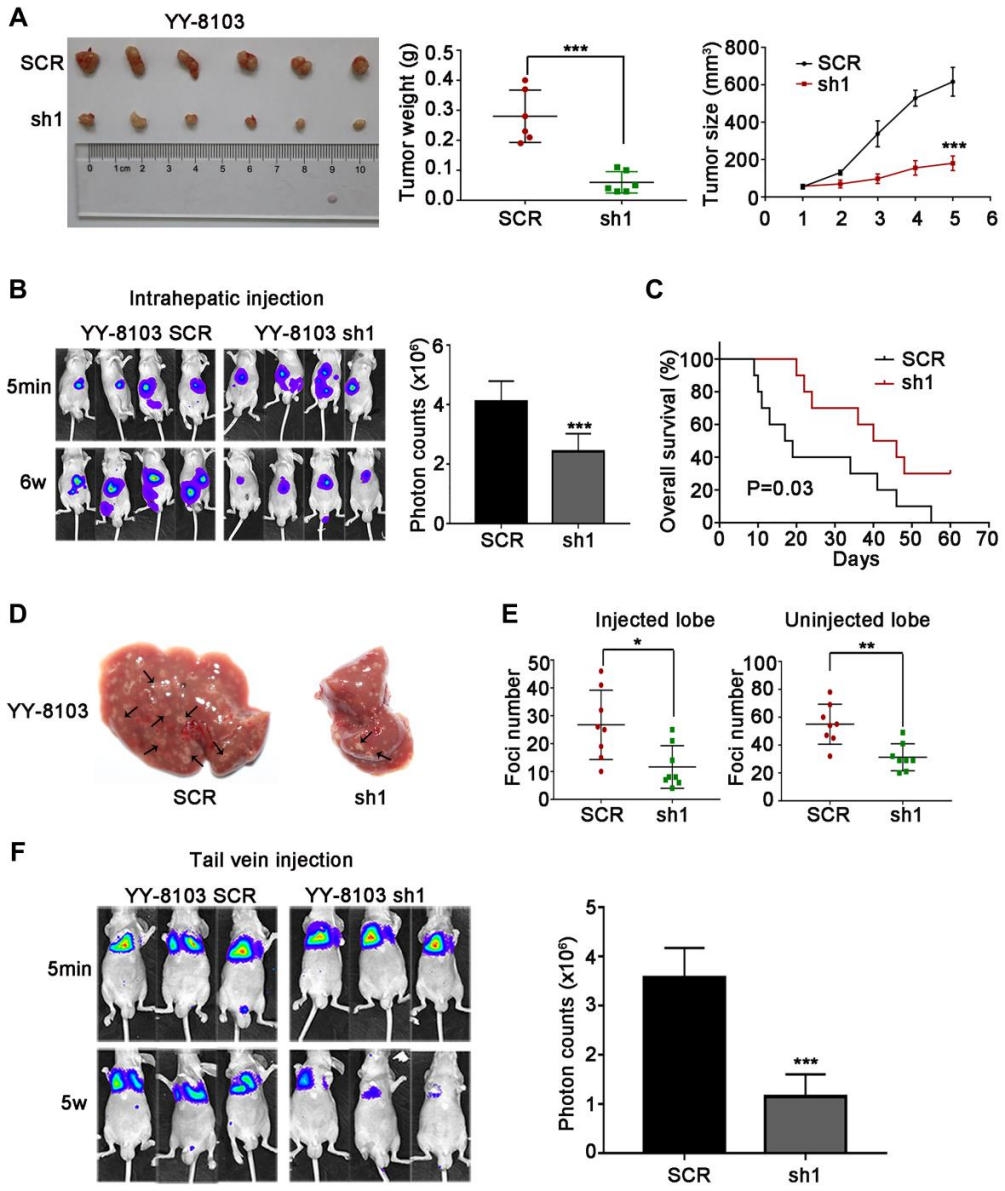
**NET1 knockdown suppresses HCC tumorigenesis, and inhibits intrahepatic and lung metastases *in vivo*.** (**A**) Left panel: representative images of tumors generated by control and NET1-knocked down YY-8103 cells. Middle panel: tumor weight (g). Right panel: tumor growth (mm^3^). (**B**) Left panel: representative images showing luciferase expression in intrahepatic tumors of mice from YY-8103 control and shNET1 groups. Right panel: quantification of luciferase expression in intrahepatic tumors. (**C**) Survival curves of mice in YY-8103 control and shNET1 groups. (**D**) Representative images of mice livers from YY-8103 control and shNET1 groups with intrahepatic injection. (**E**) Foci number of injected (left image) and non-injected (right image) lobes of mice with intrahepatic metastasis. (**F**) Left image: representative images showing luciferase expression from metastasized lungs of mice from YY-8103 control and shNET1 groups. Right image: quantification of luciferase expression in metastasized lungs. All data are presented as mean ± SE. ^*^P < 0.05, ^**^P < 0.01, ^***^P < 0.001 vs SCR cells.

**Figure 5:**
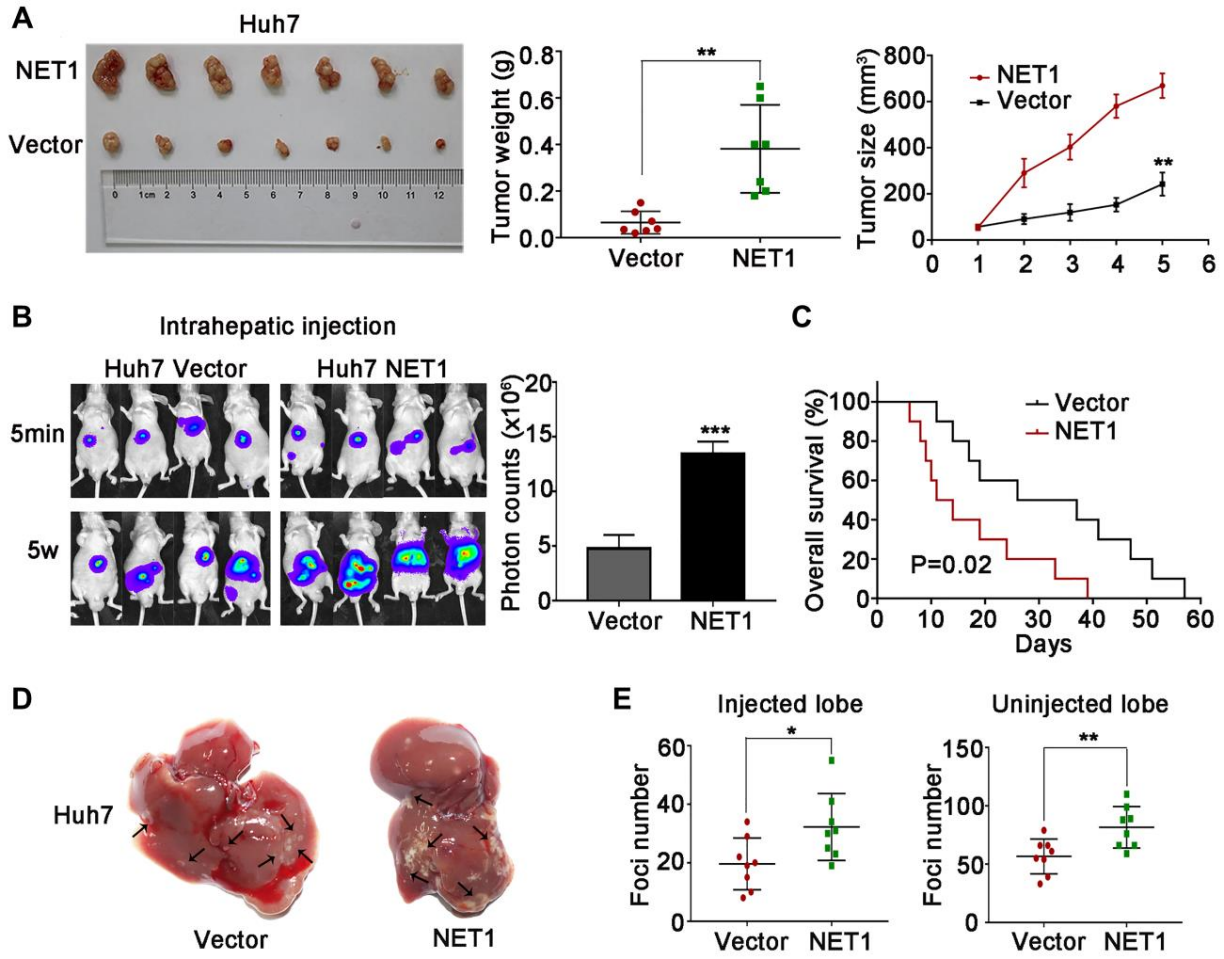
**NET1 overexpression promotes HCC tumorigenesis and intrahepatic metastases *in vivo*.** (**A**) Left image: representative images of tumors generated by control and NET1-overexpressed Huh7 cells. Middle image: tumor weight (g). Right panel: tumor growth (mm^3^). (**B**) Left panel: representative images showing luciferase expression in intrahepatic tumors of mice from control and NET1-overexpressed groups. Right panel: quantification of luciferase expression in intrahepatic tumors. (**C**) Survival curves of mice in Huh7 control and NET1-overexpressed groups. (**D**) Representative images of mice livers from Huh7 control and NET1-overexpressed groups with intrahepatic injection. (**E**) Foci number of injected (left panel) and non-injected (right photograph) lobes of mice with intrahepatic metastasis. All data are presented as mean ± SE. ^*^P < 0.05, ^**^P < 0.01, ^***^P < 0.001 vs vector cells.

The cell experiments above suggest that NET1 may induce HCC tumorigenesis and metastasis *in vivo*. To verify this hypothesis, we firstly established orthotopic xenograft assays by subcutaneously injecting NET1 knockdown and normal YY-8103 cells into both flanks of nude mice (*n* = 6 for each group). As shown in Figure 4A, knockdown of NET1 inhibited tumor growth in mice within 5 weeks of monitoring period. Moreover, the proliferation rate and masses of the tumors in NET1 silencing group were noticeably lower than those in normal group (Figure 4B, 4C). In contrast, NET1-overexpressed Huh7 cells remarkably promoted tumorigenesis in the same model (Figure 5A).

Next, we established intrahepatic metastasis assays by injecting luciferase-labelled NET1 knockdown and normal YY-8103 cells into the left lobe of each nude mouse (*n* = 8 per group). As demonstrated in Figure 4B, the luciferase signals of the mouse liver in NET1 silencing group were markedly reduced compared to normal group. Also, less tumor foci was detected in the infused/non-infused lobes of NET1 silencing mice compared to normal mice (Figure 4D, 4E). On the contrary, NET overexpression in Huh7 cells remarkably promoted intrahepatic metastasis in the same model (Figure 5B–5E).

To establish tail vein metastasis assays, control and NET1 knockdown luci-YY-8103 cells were infused into the tail vein of each nude mouse (*n* = 6 per group). Notably, the intensities of luciferase signals were markedly lower in the lungs of NET1 silencing mice than in normal mice (Figure 4F). Since high NET1 expression was observed to confer poor clinical survival, the impacts of NET1 on the survival outcomes of HCC-bearing mice (*n* = 10 for each group) were evaluated. Kaplan–Meier survival plot showed that the mice in NET1 silencing group had markedly lower survival compared to those in normal group (*P* = 0.03; Figure 4C). Conversely, the mice in Huh7 overexpression group exhibited longer survival than those in the normal expression group (*P* = 0.02; Figure 5C). Overall, these results indicate that knockdown or overexpression of NET1 play major roles in regulating HCC cell metastasis *in vivo*.

### NET1 activates Akt pathway in HCC cells

**Figure 6:**
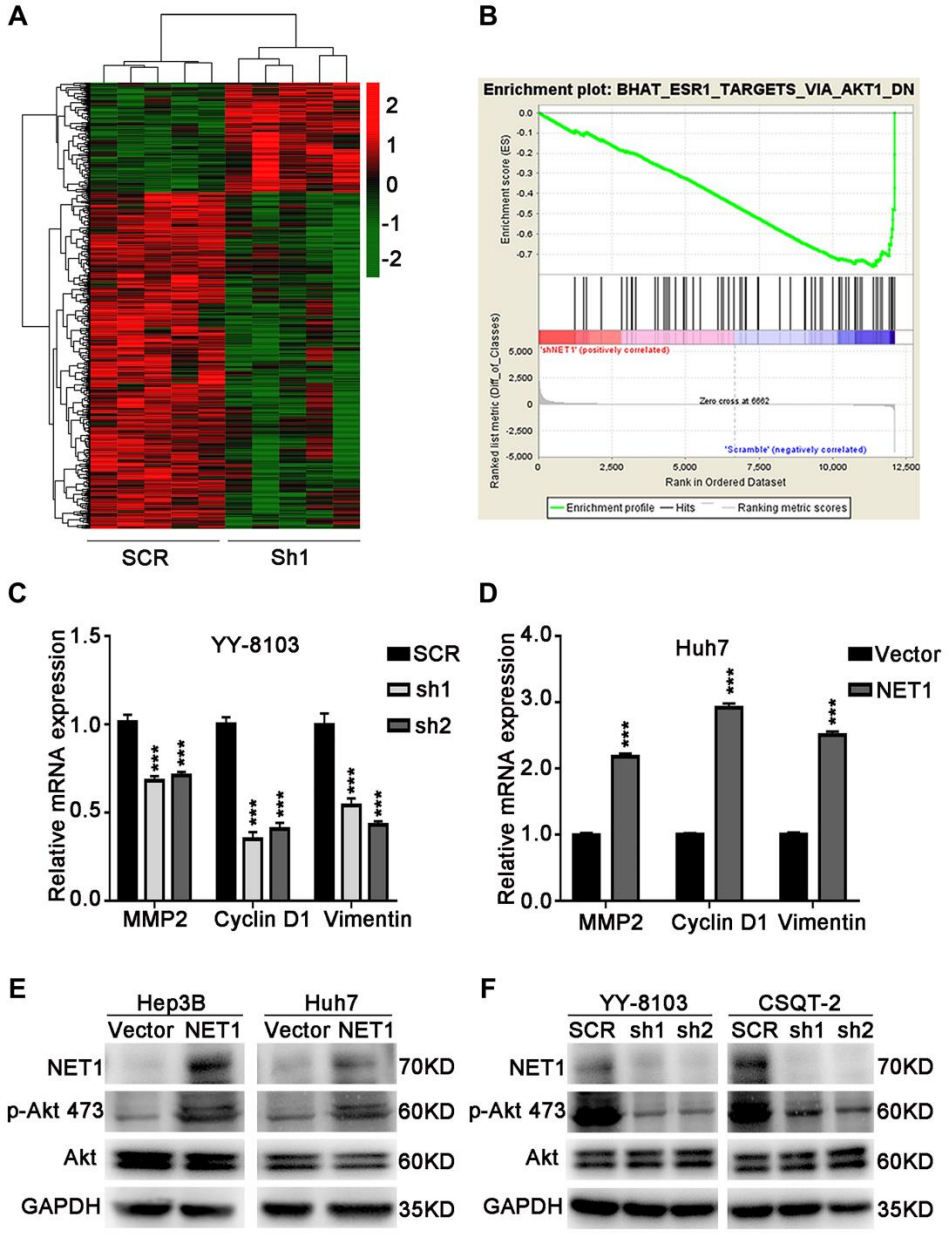
**NET1 activates Akt signaling in HCC cells.** (**A**) Hierarchical clustering generated from the expression profiles of differentially expressed mRNA levels in YY-8103 control and shNET1 groups. (**B**) Gene set enrichment analysis of altered signaling pathways in YY-8103 control and shNET1 groups. (**C**) Effects of NET1 knockdown on the expression of MMP2, Cyclin D1 and Vimentin in YY-8103 cells examined by real-time PCR. ^***^P < 0.001 vs SCR cells. (**D**) Effects of NET1 overexpression on the expression of MMP2, Cyclin D1 and Vimentin in Huh7 cells examined by real-time PCR. ^***^P < 0.001 vs vector cells. (**E**) Effects of NET1 overexpression on the phosphorylation of Akt in Hep3B and Huh7 cells examined by Western blot. (**F**) Effects of NET1 knockdown on the phosphorylation of Akt in YY-8103 and CSQT-2 cells examined by Western blot. All data are presented as mean ± SE.

To identify the underlying molecular mechanism of NET1-induced effects in HCC, RNA-seq was performed using YY-8103 control cells and NET1 knockdown pool. A Hierarchical clustering generated from the expression profiles of differentially expressed mRNA from NET1-knocked down YY-8103 cells and control cells showed distinct mRNA expression patterns between the two groups (Figure 6A). In addition, the expression of Akt signaling-associated genes was also downregulated according to GSEA analysis (Figure 6B).

As displayed in Figure 6C, the expression levels of some genes downstream of Akt, such as MMP2, Cyclin D1 and Vimentin, were dramatically decreased upon knockdown of NET1 in YY-8103 cells. Conversely, overexpression of NET1 in Huh7 cells significantly elevated the expression levels of these genes (Figure 6D). Next, we examined Akt activation by Western blotting. As presented in Figure 6E, the levels of p-Akt (Ser473) were dramatically increased in NET1-overexpressing Hep3B and Huh7 cells. In contrast, the levels of Akt Ser473 phosphorylation were remarkably decreased in NET1-knocked down YY-8103 and CSQT-2 cells compared to normal cells (Figure 6F). Altogether, these findings demonstrate that NET1 activates Akt phosphorylation in the Akt signaling pathway.

### MK2206, a potent Akt inhibitor, inhibits Akt phosphorylation and reverses NET1 overexpression-induced HCC phenotype

**Figure 7:**
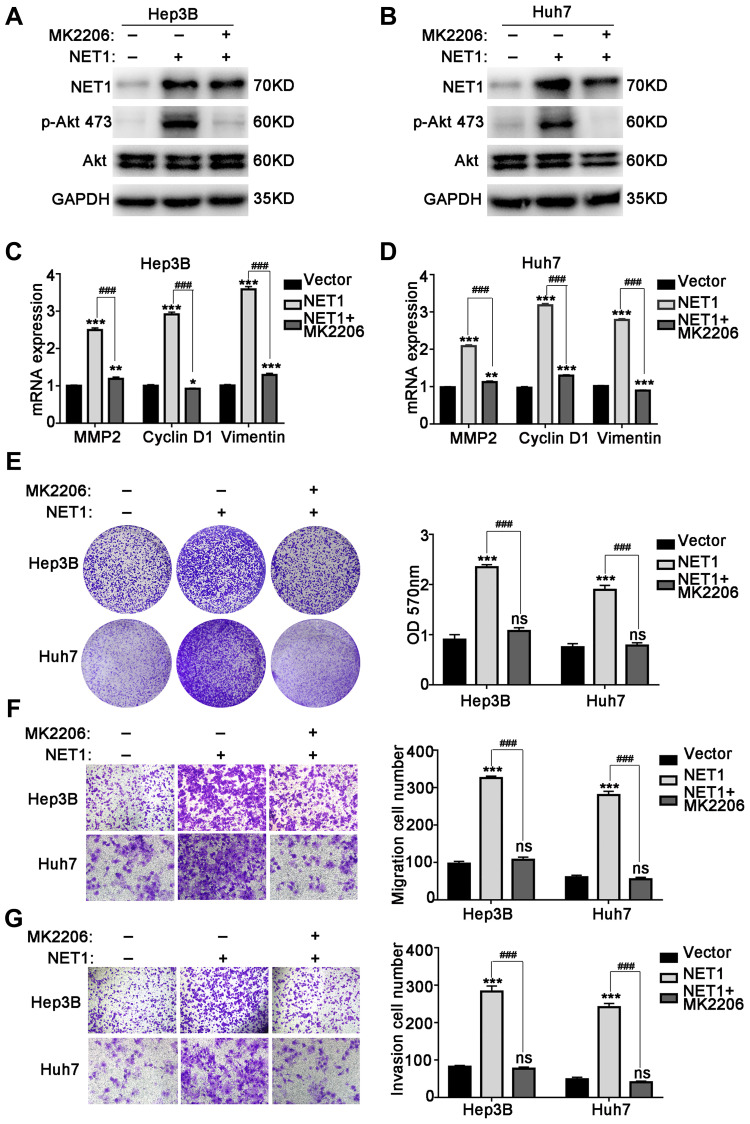
**MK2206 inhibits Akt phosphorylation and reverses NET1 overexpression-induced HCC phenotype.** (**A** and **B**) Western blot showing the inhibition of Akt phosphorylation by MK2206 in Hep3B (**A**) and Huh7 (**B**) cells with NET1 overexpression. (**C** and **D**) Real-time PCR showing the reversal of NET1-induced increased expression of MMP2, Cyclin D1 and Vimentin by MK2206 in Hep3B (**C**) and Huh7 (**D**) cells. (**E**) Crystal violet assays showing the reversal of NET1-induced cell growth by MK2206. Left panel: crystal violet assays. Right panel: measured OD values. (**F**) Transwell assays showing the reversal of NET1-induced cell migration by MK2206. Left panel: transwell assays. Right panel: calculation of cells that have migrated through the filter following eosin staining. (**G**) Transwell assays showing the reversal of NET1-induced cell invasion by MK2206. Left panel: transwell assays. Right panel: calculation of cells that have invaded through the filter following eosin staining. All data are presented as mean ± SE. ns not significant. ^**^P < 0.01, ^***^P < 0.001 vs vector cells. ^###^*P* < 0.001 vs Lenti-NET1 cells.

In addition, we determined whether the observed effect of NET1 on HCC progression is of Akt-dependent. To confirm whether the activation of Akt pathway can be blocked to reverse the NET1-induced HCC phenotype, MK2206, a potent Akt inhibitor, was incubated with NET1-overexpressed Hep3B and Huh7 cells. As demonstrated in Figure 7A and 7B, MK2206 suppressed the phosphorylation of Akt Ser473 in NET1-overexpressed Hep3B and Huh7 cells. We further demonstrated that the mRNA levels of MMP2, Cyclin D1 and Vimentin in NET1-overexpressed HCC cells were significantly lowered by MK2206 (Figure 7C, 7D). As revealed in Figure 7E–7G, the proliferation, migration and invasion of HCC cell lines induced by NET1 expression were successfully reversed through the use of MK2206. Collectively, these findings suggest that the NET1-induced effects are Akt-dependent.

## DISCUSSION

Firstly reported as an oncogene in neuroepithelial cells, NET1 plays a fundamental role in cell motility and extracellular matrix invasion [10, 11]. Similar to the results reported previously [20], this study revealed that NET1 is overexpressed in HCC tissue compared to adjacent liver tissue. HCC cases with high NET1 expression were observed to have poor clinical survival than those with low NET1 expression. Furthermore, NET1 expression was closely related to some clinical features of HCC cases, including AFP, tumor size, micro metastasis, microvascular invasion and PVTT, which are the most important clinical markers in the progression of HCC [21, 22]. Our clinical results imply that NET1 plays a promoting role in HCC progression.

In gastric tumor, NET1 has been shown to trigger cancer cell migration, invasion and cytoskeletal actin organization [12]. By analyzing the biological roles of NET1 in HCC progression, we demonstrated that knockdown or overexpression of NET1 could suppress or induce the proliferation, migration and invasion of HCC cells, respectively. Furthermore, through orthotopic xenograft assays, intrahepatic metastasis assays and lung metastasis assays, we showed that NET1 plays a role in mediating HCC tumorigenesis and metastasis *in vivo*.

Akt has been shown to affect various downstream effectors and regulate many pathways that promote cell growth, invasion and migration [23–27]. Furthermore, Akt is a common protein kinase that can be hyperactivated in human tumors [28]. Akt is activated by nearly all known angiogenic factors, cytokines and growth factors. Consistently, the important constituents of Akt pathway are shown to be amplified or mutated in many cancer types [29, 30]. However, the association between Akt and NET1 has not been reported. In this study, we performed RNA-seq to obtain a comprehensive understanding of NET1 signaling. Our GSEA analysis demonstrated that Akt1 pathway may be associated with the function of NET1 in HCC. NET1 was found to regulate the mRNA levels of Akt1 downstream genes (cyclin D1, MMP2 and vimentin) that are associated with cell growth, invasion or migration in HCC cells. Furthermore, we found that depletion of NET1 expression significantly suppresses the level of AKT phosphorylation and that, NET1 overexpression remarkably promotes the level of AKT phosphorylation in HCC cells. MK2206, an allosteric inhibitor of Akt, was used to reverse the NET1 overexpression-induced HCC cell phenotype. We demonstrated that MK2206 inhibits HCC growth, invasion and migration by inhibiting the levels of NET1 overexpression-induced AKT phosphorylation and protein expression of Akt downstream genes in HCC cells. In conclusion, this study demonstrates the relationship between NET1 and HCC progression, particularly tumor growth, invasion and metastasis, through the activation of Akt1 signaling pathway. Our results also suggest that Akt inhibitor may be beneficial to HCC cases with NET1 overexpression.

In summary, novel roles of NET1 in HCC cell proliferation and metastasis were illustrated through *in vivo* and *in vitro* approaches. Our study offers a new insight and comprehensive understanding of the mechanisms of NET1 in HCC development. Our findings imply that NET1 can serve as a promising treatment target for HCC.

## Supplementary Materials

Supplementary Tables
