# Supplementary material for: NET1 promotes HCC growth and metastasis in vitro and in vivo via activating the Akt signaling pathway
Source: Aging (Albany NY). 2021 Apr 11;13(7):10672–87. doi: 10.18632/aging.202845 (PMC8064201; doi:10.18632/aging.202845)
Supplement: Supplementary Tables [file aging-13-202845-s001.pdf]

SUPPLEMENTARY TABLES

Supplementary Table 1. Primer sequence.

Gene	Forward	Reverse
NET1	TGCTCTAGAATGGAGCCCGAGCTGGCGGCTC AGA	CCATCGATCCACCAAAGTCTCTTTCCGTTTGC CA
NET1-Sh1	CCGGCCCGAGGTGAACAGGATTTAACTCGAG TTAAATCCTGTTCACCTCGGGTTTTTG	AATTCAAAAACCCGAGGTGAACAGGATTTAA CTCGAGTTAAATCCTGTTCACCTCGGG
NET1-Sh2	CCGGCGCCTAGTCAAATACCCTTTACTCGAG TAAAGGGTATTTGACTAGGCGTTTTTG	AATTCAAAAACGCCTAGTCAAATACCCTTTA CTCGAGTAAAGGGTATTTGACTAGGCG
MMP2	CCCACTGCGGTTTTCTCGAAT	CAAAGGGGTATCCATCGCCAT
Cyclin D1	GCTGCGAAGTGGAAACCATC	CCTCCTTCTGCACACATTTGAA
Vimentin	GACGCCATCAACACCGAGTT	CTTTGTCGTTGGTTAGCTGGT

Supplementary Table 2. Correlation between NET1 expression and clinicopathological characteristics.

Variables	Number	NET1, *n* (%)		*P*-value
		Low expression	High expression	
Age, years				0.890
<50	107	53 (50.5%)	54 (51.4%)	
≥50	103	52 (49.5%)	51 (48.6%)	
Sex				0.507
Male	187	92 (87.6%)	95 (90.5%)	
Female	23	13 (12.4%)	10 (9.5%)	
HBsAg				0.831
Negative	25	13 (12.4%)	12 (11.4%)	
Positive	185	92 (87.6%)	93 (88.6%)	
AFP, ng/ml				**0.015**
<400	133	75 (71.4%)	58 (55.2%)	
≥400	77	30 (28.6%)	47 (44.8%)	
Total bilirubin, μmol/l				0.482
≤17.1	170	87 (82.9%)	83 (79.0%)	
>17.1	40	18 (17.1%)	22 (21.0%)	
Albumin, g/l				0.580
<35	14	6 (5.7%)	8 (7.6%)	
≥35	196	99 (94.3%)	97 (92.4%)	
ALT, U/L				0.478
≤44	129	62 (59.0%)	67 (63.8%)	
>44	81	43 (41.0%)	38 (36.2%)	
Tumor diameter, cm				**0.011**
<5	65	41 (39.0%)	24 (22.9%)	
≥5	145	64 (61.0%)	81 (77.1%)	
Number of tumors				0.675
Single	184	93 (88.6%)	91 (86.7%)	
Multiple	26	12 (11.4%)	14 (13.3%)	
Micro metastasis				**0.004**
Negative	130	75 (71.4%)	55 (52.4%)	
Positive	80	30 (28.6%)	50 (47.6%)	
PVTT				**0.025**
Negative	176	94 (89.5%)	82 (78.1%)	
Positive	34	11 (10.5%)	23 (21.9%)	

Microvascular invasion				**0.025**
Negative	122	69 (65.7%)	53 (50.5%)	
Positive	88	36 (34.3%)	52 (49.5%)	

Abbreviation: HBsAg: Hepatitis B surface antigen; AFP: α-fetoprotein; ALT: Alanine Aminotransferase; PVTT: portal vein tumor thrombus. *P* < 0.05 is shown in bold.

Supplementary Table 3. Univariate and multivariable analysis for overall survival.

Variables	Univariate analysis		Multivariate analysis	
	HR (95%CI)	*P*-value	HR (95%CI)	*P*-value
Age, ≥50 years	1.009 (0.667–1.527)	0.965		
Sex, male	1.472 (0.712–3.043)	0.297		
HBsAg, positive	1.175 (0.590–2.340)	0.647		
AFP, ≥400 ng/ml	2.588 (1.699–3.941)	**<0.001**	2.151 (1.353–3.419)	**0.001**
Total bilirubin, >17.1 µmol/l	1.403 (0.852–2.308)	0.183		
Albumin, ≥35 g/l	0.525 (0.253–1.090)	0.084		
ALT, >44 U/L	1.092 (0.714–1.668)	0.685		
Tumor diameter, ≥5 cm	3.087 (1.790–5.321)	**<0.001**	2.017 (1.106–3.577)	**0.022**
Number of tumors, multiple	3.300 (1.954–5.575)	**<0.001**	3.073 (1.742–5.423)	**<0.001**
Micro metastasis, positive	2.620 (1.722–3.987)	**<0.001**	1.279 (0.780–2.098)	0.329
PVTT, positive	2.708 (1.636–4.483)	**<0.001**	1.579 (0.907–2.749)	0.106
Microvascular invasion, positive	3.517 (2.289–5.403)	**<0.001**	2.116 (1.286–3.481)	**0.003**
NET1, high expression	2.319 (1.495–3.596)	**<0.001**	1.926 (1.200–3.092)	**0.007**

Abbreviation: HBsAg: Hepatitis B surface antigen; AFP: α-fetoprotein; ALT: Alanine Aminotransferase; PVTT: portal vein tumor thrombus. *P* < 0.05 is shown in bold.

Supplementary Table 4. Univariate and multivariable analysis for disease-free survival.

Variables	Univariate analysis		Multivariate analysis	
	HR (95%CI)	*P*-value	HR (95%CI)	*P*-value
Age, ≥50 years	1.075 (0.741–1.550)	0.704		
Sex, male	1.361 (0.730–2.538)	0.332		
HBsAg, positive	1.229 (0.659–2.293)	0.516		
AFP, ≥400 ng/ml	1.981 (1.356–2.894)	**<0.001**	1.498 (0.988–2.248)	0.051
Total bilirubin, >17.1 µmol/l	1.143 (0.722–1.810)	0.568		
Albumin, ≥35 g/l	0.724 (0.352–1.492)	0.382		
ALT, >44 U/L	1.458 (0.998–2.130)	0.051		
Tumor diameter, ≥5 cm	1.754 (1.145–2.686)	**0.010**	1.226 (0.757–1.987)	0.408
Number of tumors, multiple	1.500 (0.838–2.686)	0.172		
Micro metastasis, positive	2.414 (1.656–3.517)	**<0.001**	1.765 (1.141–2.731)	**0.011**
PVTT, positive	2.497 (1.581–3.943)	**<0.001**	1.536 (0.935–2.522)	0.090
Microvascular invasion, positive	2.032 (1.390–2.969)	**<0.001**	1.335 (0.851–2.094)	0.209
NET1, high expression	2.222 (1.499–3.294)	**<0.001**	1.896 (1.246–2.883)	**0.003**

Abbreviation: HBsAg: Hepatitis B surface antigen; AFP: α-fetoprotein; ALT: Alanine Aminotransferase; PVTT: portal vein tumor thrombus. *P* < 0.05 is shown in bold.
